# Moral distress thermometer: Swedish translation, cultural adaptation and validation

**DOI:** 10.1177/09697330231197707

**Published:** 2023-09-27

**Authors:** Catarina Fischer Grönlund, Ulf Isaksson, Margareta Brännström

**Affiliations:** 8075 Umeå University

**Keywords:** moral distress, health care professionals, questionnaire, instrument, translation, validation

## Abstract

**Background:**

Moral distress is a problem and negative experience among health-care professionals. Various instruments have been developed to measure the level and underlying reasons for experienced moral distress. The moral distress thermometer (MDT) is a single-tool instrument to capture the level of moral distress experienced in real-time.

**Aim:**

The aim of this study was to translate the MDT and adapt it to the Swedish cultural context.

**Research design:**

The first part of this study concerns the translation of MDT to the Swedish context, and the second part the psychometric testing of the Swedish version.

**Participants and research context:**

89 healthcare professionals working at a hospital in northern Sweden participated. Convergent validity was tested between MDT and Measure of Moral Distress-Healthcare Professionals (MMD-HP), and construct validity was tested by comparing MDT scores among healthcare professionals. MDT was compared with responses to the final questions in MMD-HP. One-way ANOVA, Welch’s ANOVA, Games–Howell post-hoc test and Pearson’s correlation analysis were done.

**Ethical considerations:**

The study was approved by the Swedish Ethics Review Authority (dnr 2020-04120) in accordance with Helsinki Declaration.

**Results:**

The translated Swedish version of MDT was described as relevant to capture the experience of moral distress. The mean value for MDT was 2.26, with a median of 2 and a mode value of 0. The result showed moderate correlations between the MDT and MMD-HP total scores. There was a significant difference when comparing MDT and healthcare professionals who had never considered leaving their present position with those who had left and those who had considered leaving but had not done so, with the latter assessing significantly higher moral distress.

**Conclusion:**

The MDT is an easily available instrument useful as an extension to MMD-HP to measure the real-time experience of moral distress among healthcare professionals in a Swedish context.

## Introduction

Various instruments have been developed to measure the level and underlying reasons for experienced moral distress. This study is a part of the research project ‘Ethics Com Study’, with the primary purpose of measuring the effectiveness of an intervention with interprofessional ethics communication in groups in health care. One part of the study entails measuring the pre- and post-experience of moral distress using the Measure of Moral Distress for Healthcare Professionals (MMD-HP) and The Moral Distress Thermometer (MDT) questionnaires.

## Background

Moral distress has been described as a severe problem in healthcare and as a negative experience among various professional groups.^1–3^ Jameton^4^ originally defined the concept of moral distress as a condition of frustration, guilt and anger due to feeling prevented from giving care according to one’s values about what is right and good. Hamric^5^ has defined moral distress as a condition that ‘occurs when an individual’s moral integrity is seriously compromised, either because one feels unable to act in accordance with core values and obligations, or attempted actions fail to achieve the desired outcome’.

Moral distress can be linked to various fundamental causes, such as a defensive and unsupportive organisational culture,^6^ professional uncertainty about moral judgements,^7^ or lack of moral integrity or courage to stand up for one’s convictions.^6^ Epstein et al.^8^ found root causes for moral distress at system level, clinical causes, patient level and team level, with a differentiation between integrity occurring within a team or breakdowns in the team’s interactions with patients and families.^8^

The experience of moral distress has been studied among professionals from different healthcare contexts, such as community healthcare,^1^ oncology care,^9^ intensive care^10^ and paediatric care.^11^ Various instruments to measure the experience of moral distress have been developed, such as the Ethical Distress Scale, Moral Distress Intensity Scale, Moral Distress Risk Scale (MDRS)^12^ and the Moral Distress Scale (MDS), initially developed by Corley et al.^13^ The instrument was shortened by Hamric and Blackhall,^14^ revised to the Moral Distress Scale-Revised (MDS-R), and extended to include healthcare professionals from outside intensive care.^15^ There are six versions of the MDS-R aimed at; adult physicians, adult nurses and adult other healthcare professionals, paediatric physicians, paediatric nurses and other paediatric health care professionals.^8^ The MDS-R was further slightly modified in order to suit additional clinicians^16^ and revised into the MMD-HP.^8^ The reason was to more fully capture system-level and team-level root causes of moral distress and underlying reasons for the experience of moral distress among healthcare workers from various professions and contexts.^8^ The MDS-R and MMD-HP instruments are intended to measure two aspects of moral distress: frequency and intensity.^8,15^ The MMD-HP has been translated into Japanese,^17^ Spanish^10^ and Swedish.^18^

The MDT was developed by Wocial and Weaver^19^ as a single-tool instrument to catch the level of moral distress experienced in real-time. The MDT has been used to measure moral distress among healthcare professionals worldwide, for instance, in critical care,^20^ Italian community and hospital care,^1^ in Australian neonatal care,^11^ among members of the American College of Physicians^21^ and school nurses in North Carolina, USA^22^; and in German hospitals^23^ and oncology care.^9^

The MMD-HP, previously translated, culturally adapted^18^ and validated for the Swedish context^24^ is designed to measure root causes and the overall experiences of moral distress.^8,25^ The MMD-HP instrument was developed to measure moral distress among healthcare professionals from various professions and contexts and therefore found to be most useful for the ‘Ethics Com Study’ aim. MDT can be seen as a complement to the MMD-HP. Hence, this single-item scale can rapidly assess and rate the experience of moral distress.^25^ However, to our knowledge, the instrument has not been used in a Swedish context. Therefore, the aim was to translate, culturally adapt and validate the moral distress thermometer in the Swedish context.

## Methods

### The moral distress thermometer

The MDT developed by Wocial and Weaver^19^ is a single-tool instrument. It includes a rating scale from 0 to 10, where every second degree is associated with a descriptive word related to the degree of moral stress. The descriptive wordings rate from none, mild, uncomfortable, distressing, intense and worst possible levels of moral distress. The instrument asks the respondent to reflect on the level of moral distress experienced during the past 2 weeks.

The original version includes the following introduction: ‘Moral distress occurs when you believe you know the ethically correct thing to do, but something or someone restricts your ability to pursue the right course of action. Please circle the number (0–10) on the thermometer that best describes how much moral distress you have been experiencing related to work in the past 2 weeks, including today’.^19^ Construct validity of the original version of MDT compared to the MDS 2009 resulted in a low to moderate correlation between the instruments.^19^ This study is in two parts: the first part concerns the translation process to the Swedish context, and the second part the psychometric testing of the Swedish version of MDT, which this paper describes.

### The measure of moral distress-healthcare professionals

The Measure of Moral Distress-Healthcare Professionals (MMD-HP)^8^ was used to test for construct validity. The questionnaire consists of 27 items of ethically difficult situations or dilemmas in healthcare. The items are assessed on intensity and frequency on a five-point Likert scale from non-to very distressing and never to very frequent. In addition, the MMD-HP includes two questions regarding whether a person has left or intends to leave their position because of moral distress. A total score is calculated by multiplying each item’s frequency and intensity level, giving an overall score ranging from 0 to 432. The MMD-HP also consists of four sub-scales: System level, Clinical causes, Team/staff and Team/patient.^8^

### Translation of the moral distress thermometer

The designer of the MDT, Lucia D. Wocial, permitted its translation from English into Swedish. The translation followed the WHO^26^ guidelines and a four-step process: forward translation, back translation, expert panel scrutiny and pre-testing/cognitive interviewing. The MDT was also adapted to the Swedish cultural context during the translation process.

### Forward translation

The original version of the MDT was translated into Swedish in two versions by two native Swedish healthcare professionals with extensive English language experience. One translator was a physician in emergency and cardiac medicine; the other was a nurse assistant in elderly care; they translated the instrument independently. In addition, the first and third authors reviewed and merged the two versions of the forward translation.

### Back translation

The authors discussed differences in word choice in the two versions and decided on the final formulations. Two native English translators did back translation of the instrument from Swedish into English with extensive Swedish knowledge. The back translators had not seen the original version of the questionnaire, and they worked independently from each other. The authors reviewed the back-translated versions. Words were analysed according to meaning and content and compared with the pre-translated version.

### Expert panel

The expert panel for content validation of the back-translated version consisted of members (*n* = 7) from the research group: two professors, two associate professors and three assistant professors. First, the researchers reviewed the back translation of the Moral Distress Thermometer and compared it with the pre-translated version. In addition, cognitive interviews were carried out individually (*n* = 2) or in groups (*n* = 5). Finally, every word in the scale was discussed concerning comprehensibility, relevance and definition clarity.

### Pre-testing and cognitive interviews

Cognitive face-to-face interviews were performed with healthcare professionals from various clinical areas at the hospital (*n* = 10) to achieve respondent satisfaction, face validity and content validity. The professionals comprised two males and eight females, aged 31–75 years and 7–47 years of professional experience. They were working in various Swedish clinical contexts as an occupational therapist [OT] (*n* = 1), a physiotherapist [PT] (*n* = 1), a physician (*n* = 1), registered nurses [RNs] (*n* = 4), enrolled nurses [ENs] (*n* = 2) and an occupational therapist (*n* = 1).

The authors performed the interviews individually in a digital meeting room (*n* = 8) or a separate room (*n* = 2). The participants got access to the translated version of the Moral Distress Thermometer in time for the interview. The think-aloud method was used for the interviews;^27^ hence, the participants were asked to reflect on each choice of wording on the thermometer scale according to relevance, comprehensibility, and clarity and to suggest alternative wordings. The participants read and voiced the translated wording choices on the scale, expressed the meaning or concern of the words according to the scale, and suggested alternatives. Written notes were made during the interview, and the authors asked further clarifying questions when needed.

Throughout the translation process, the ambition was to keep as close as possible to the original formulations. Therefore, the cognitive interviews with the healthcare professionals focused on relevance and clarity/comprehensibility of the forward, back-translated and first-reviewed descriptive wordings of the MDT rating scores, resulting in the final descriptive wordings.

#### Relevance

In general, the translated Swedish version of MDT was described as relevant to capture the experience of moral distress. Also, the rating scores on the thermometer were found to be relevant. According to their gradings, the interviewees found the descriptive wordings relevant.

*‘No problems with the wordings, relevant ranking’*.

#### Clarity/comprehensibility

Aspects related to comprehensibility/clarity concerned the understandability of the words. The participants described the introduction as straightforward and easy to understand. They could understand how to use the thermometer and indicate their moral distress levels. In addition, descriptive wordings were found to clarify the MDT rating scores.

*‘A thermometer is good, and the definition [on the scale axis] clarifies the rating*’.

Moral distress as a concept is related to burdensome emotions of frustration, anguish and threatened moral integrity. However, no everyday Swedish word comprises that concept. Therefore, moral stress was the most useful term to convey the meaning in everyday language.

### Study setting and participants for validation

Healthcare professionals working in nine departments at a hospital in northern Sweden were given oral and written information and were invited to participate in the study. The departments specialised in emergency care, critical care, infection care, thoracic care, palliative care, neurorehabilitation and geriatric care. Healthcare workers (*n* = 89) from various professions agreed to participate. However, respondents with more than 10% missing values from the MMD-HP questionnaire were excluded from the analysis. Hence, in the final analysis, the number of respondents dropped from 89 to 80.

### Data collection

The questionnaire, including the MDT and MMD-HP, was distributed to the participants by the head or ethical representative of the department. The questionnaires were returned in a sealed envelope and collected by the first researcher.

### Statistics

We tested for convergent validity by means of correlation coefficients between the total score and the four different sub-scales in the MMD-HP developed by Epstein et al.^8^ Pearson’s correlation coefficient was used since we assumed that MDT data was not at an interval level but more at the ordinal level. Construct validity was tested by comparing MDT scores among healthcare professionals in different settings, such as emergency department, geriatric care, intensive care, infection care, neurological care and palliative home care. We expected that moral distress would be roughly equivalent between these activities unless something specific occurred that posed an ethical problem or dilemma for healthcare professionals. Furthermore, MDT was compared with the following responses to the final questions in MMD-HP, ‘Have you ever left or considered leaving a clinical position due to moral distress’? – ‘No: I have never considered leaving or left a position’, ‘Yes: I considered leaving but did not leave’ and ‘Yes: I left a position’.

A one-way ANOVA was used for these comparisons, and a Welch’s ANOVA was used if a homogeneity violation was found. In addition, a Games–Howell post-hoc test was used. Furthermore, Pearson’s correlation analysis was done to analyse correlations between the MDT and MMD-HP. JAMOVI, version 2.3.18, was used in all analyses except for analysing the distribution of response options on the MDT, where Excel was used. A *p*-value <.05 was considered significant.

### Ethical considerations

The study has been performed in line with the Helsinki Declaration (2013). All participants gave their consent to participate after receiving oral and written information about the study. The participants were also informed that their participation was voluntary and that they could end participation if they wanted to without any consequences. The study was approved by the Swedish Ethics Review Authority (dnr 2020-04120).

## Result

### Demographic characteristics of the sampling

The study sample included healthcare professionals (*n* = 80) from various professions, including enrolled nurses (*n* = 23), registered nurses (*n* = 34), physicians (*n* = 7), paramedics (*n* = 12) and other professions (*n* = 4). The sample consisted of 68 women and 12 men aged between 20 and 61 years, with a median work experience within the profession of 14 (IQR = 5.0–24.5) years and at the present workplace of 6 (IQR = 2.0–12.3) years.

**Figure 1.**
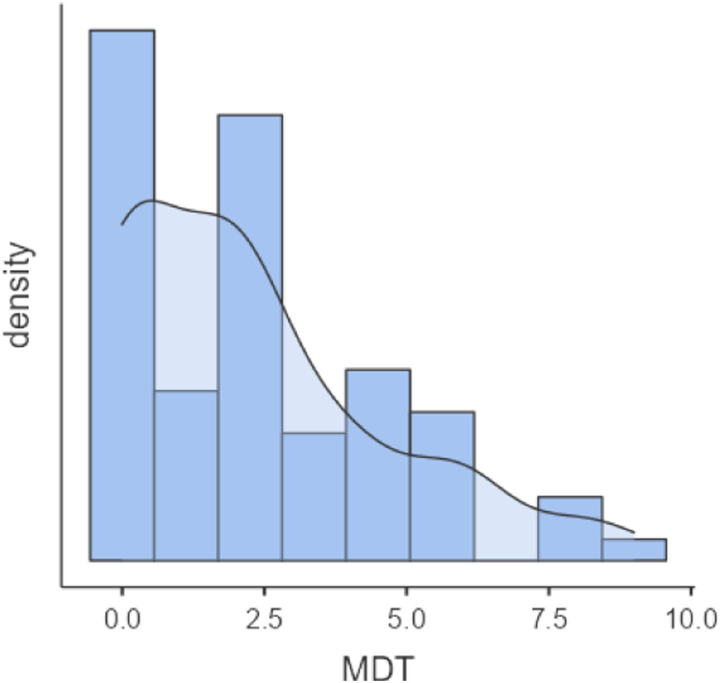
Distribution of MDT scores.

**Figure 2.**
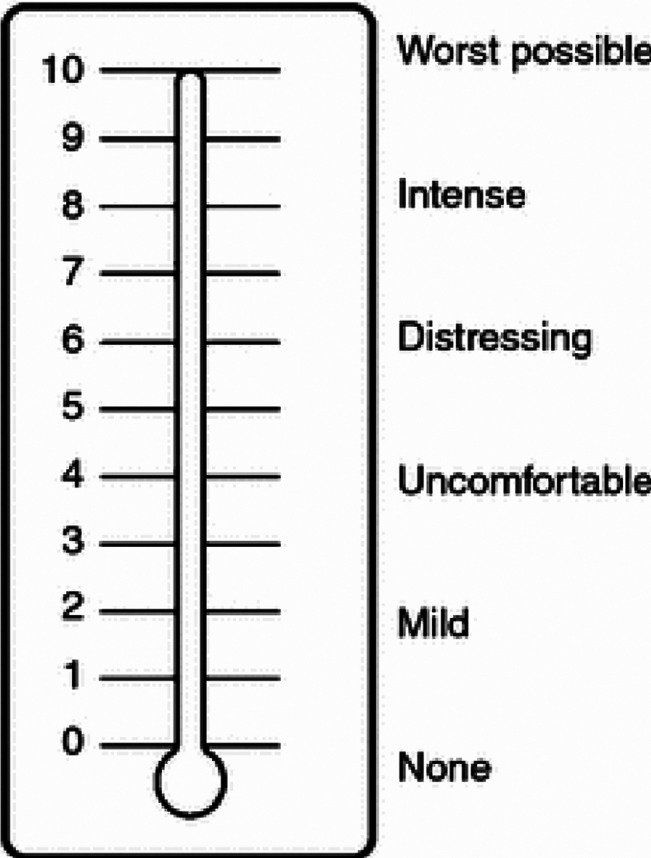
The moral distress thermometer with the rating of perceived moral stress to the left side and number of respondents answers to the right cf^19^. This figure was developed by Dr. Lucia Wocial and has the copyright of Moral distress Therometer image. Permission has been received to use this image.

The mean value for MDT was 2.26 (±2.32, 95% CI 1.75–2.78) and a median of 2 (IQR = 0.0–3.25) with a mode value of 0. The result showed one (1) outlier assessing a score of 9. The distribution of MDT scores is shown in Figure 1. Moral distress, measured by MMD-HP, was low to moderate, with a mean of 67.81 (±42.22) of a possible 432. However, the results showed that the respondents tended to use the labelled answer options (see Figure 2).

### Convergent validity

The MMD-HP was used to determine the convergent validity of the MDT. Table 1 shows the correlations between the MDT and total score of MMD-HP and the four different sub-scales in the MMD-HP. The result shows moderate correlations between the MDT and MMD-HP total score, except for the sub-scale clinical causes.

**Table 1.** Pearson correlation coefficients for the moral distress thermometer and moral distress scale, total score and the four sub-scales in the MMD-HP.

	Moral distress thermometer
MMD-HP total score	0.50***
MMD-HP system level	0.44***
MMD-HP clinical causes	0.28*
MMD-HP team/staff	0.49***
MMD-HP team/patient	0.40***

*Note*. *p < .05, **p < .01, ***p < .001.

### Construct validity

The result showed no significant differences between professionals working in different healthcare facilities, that is, emergency department, geriatric care, intensive care, care for infectious diseases, neurological care and palliative home care (F = 1.12; df = 5, 74; *p* = .358). However, the result showed a significant difference when comparing MDT and healthcare professionals who had never considered leaving their present position with those who had left and those who had considered leaving but had not done so (F = 5.22; df = 2, 32.70; *p* = .011). In addition, Games–Howell post-hoc test showed that those who had considered leaving but had not done so assessed significantly higher than the other two groups (considered leaving = 3.73 vs never considered = 1.64, left = 1.92).

## Discussion

The MDT was translated, culturally adapted to the Swedish context and validated in this study. The WHO Organization^26^ guidelines for translation were followed through the translation process, and the ambition was to keep instructions and wordings equivalent to the original version of the MDT. According to Epstein et al.,^28^ any translated and validated version can be useful if a rigorous translation process is performed and equivalence with the original instrument version is preserved. Based on the cognitive interviews, the translated Swedish version of the descriptive part of MDT was found relevant to capture the experience of moral distress. Also, the introduction describing how to use the thermometer and the definition of moral distress was clear.

Moral distress is a burdensome condition with stress and anguish that arises when the moral integrity becomes threatened. However, the concept of moral distress is not a natural everyday definition in the Swedish language. Therefore, moral stress is a more useful term for Swedish healthcare professionals in general. The two concepts have different meanings in English, but it was considered important to make the concept understandable for all responders. Moral distress and moral stress have been described as psychological reactions derived from stress-related moral problems.^29^ Also, in previous translations and cultural adaptation of MDS-R for Swedish paediatric care^30^ and MMD-HP,^18^ moral stress was found to be the preferable concept to use.

The result showed moderate correlations between the MDT with MMD-HP total score, except for the sub-scale Clinical causes. This is opposite to a study by Wocial et al.^31^ who found clinical causes to be contributing factors for increasing levels of moral distress by using MDS-R and MDT. In comparison to MMD-HP, a questionnaire consisting of 27 items and two alternatives for responding (frequency and intensity of moral distress), the MDT is a visual analogue scale (VAS) assessing none (0) to worst possible (10) moral distress. Therefore, we argue that the MDT can be used as an extension to MMD-HP to assess the real-time experience of moral distress and as an available instrument for busy clinicians to respond to, which may increase the response rate.^25^

The result showed a significant difference between the scoring of the MDT and the final questions in the MMD-HP questionnaire concerning healthcare professionals who had never considered leaving their present position with those who had left and those who had considered leaving but had not done so. Those who had considered leaving but had not done so assessed a higher level of moral distress in MDT than the other two groups. In a study by Wolf et al.,^20^ three additional questions were used concerning participants’ intention to leave or whether they had previously left their position because of moral distress. Similar to our result, respondents who currently intended to leave their position rated significantly higher scores on the MDT than other respondents.^20^ Leaving the position seems to be one way for healthcare professionals to handle moral distress at the workplace. In a theoretical framework, Rushton et al.^32^ emphasised that if moral distress cannot be modulated, it may compromise a person’s wellbeing and cause moral harm, leading to outrage and burnout. Avoidance and abandonment can be ways to handle such suffering. Wolf et al.^20^ mean that being emotionally numb, avoiding ethically challenging situations and leaving the position can be ways for professionals to handle suffering caused by moral distress. The MDT has some response options formulated as support for rating. However, the formulations may be interpreted differently between the respondents. Nevertheless, the numbering of the VAS scale as well as the wording of the various response options works as further support by showing the direction of the moral distress level.

We argue that the MDT can be useful for measuring experienced real-time levels of moral distress and tracking changes over time, both in research and actual clinical everyday situations in Swedish healthcare. Epstein et al.^25^ used the MDT as an easily available instrument for respondents, allowing for quick estimation of real-time moral distress according to a specific situation. Furthermore, the MDT has been used to measure the outcome of various interventions related to moral distress. For example, a study by Semler^33^ showed a decreased level of moral distress after a workshop intervention using the MDT. In a study by Guttmann et al.,^34^ the level of moral distress further increased after a Goal of Care Intervention. However, Wocial et al.^31^ found no difference in the level of moral distress after an intervention with interprofessional rounds.

This study was performed in settings with a variety of clinical specialities. According to MDT, the results showed no difference in the perceived level of moral distress among professionals working in different clinical settings. However, in a study by Wocial et al.,^35^ physicians rated higher on the MDT scale when caring for hospitalised patients than those in nursing homes. Wolf et al.^20^ showed that nurses in the critical care units rated higher levels on the MDT due to a low frequency and lacking knowledge of palliative care. Moral distress has found to be higher in ICUs since sources of moral distress are more frequent and intense with conflicting goals, more infliction of harm, pain and suffering.^20^ During the pandemic, nurses from different hospital settings rated high levels of moral distress comparable to those working in critical care before the COVID-19 pandemic.^21^

One limitation of this study is that no test-retest was performed; therefore, it is difficult to be sure if the instrument is stable over time or changes depending on interventions. However, according to Tian et al.,^36^ the MDT is useful for measuring the experience of moral distress in real-time and over time. Therefore, even if we assume that it also applies to the Swedish version of the MDT, further studies must be performed with repeated measurements over time to establish reliability and validity more rigorously. Another limitation could be the choice of using MMD-HP instead of MDS-R to measure moral distress and validate the MDT; hence, the MMD-HP is used and validated to a lesser extent. Since the MMD-HP instrument was developed to measure moral distress among healthcare professionals from various professions and contexts, it was found to be the most suitable for the ‘Ethics Com Study’.

## Conclusion

We found the Swedish translated and culturally adapted version of the MDT reliable and valid for measuring moral distress among healthcare professionals in a Swedish context. We argue that the MDT is a valuable instrument as it can be completed quickly and indicates the moral distress of an individual or a group well. The Swedish version is compatible with the original version in English, but further studies with repeated measurements are needed to establish reliability and validity more rigorously.

## References

[1] Giannetta N Sergi R Villa G , et al. Levels of moral distress among health care professionals working in hospital and community settings: a cross sectional study. Healthcare 2021; 9: 1673. 34946401 10.3390/healthcare9121673 PMC8701919

[2] Fronek P Briggs L Kim M , et al. Moral distress as experienced by hospital social workers in South Korea and Australia. Soc Work Health Care 2017; 56: 667–685. 28723309 10.1080/00981389.2017.1347596

[3] Allen R Judkins-Cohn T deVelasco R , et al. Moral distress among healthcare professionals at a health system. JONA’s Healthc Law, Ethics, Regul 2013; 15: 111–118. 23963112 10.1097/NHL.0b013e3182a1bf33

[4] Jameton A . Dilemmas of moral distress: moral responsibility and nursing practice. Clin Issues Perinat Womens Health Nurs 1993; 4: 542–551. 8220368

[5] Hamric A . A case study of moral distress. J Hospice Palliat Nurs 2014; 16: 457–463.

[6] Gallagher A . Moral distress and moral courage in everyday nursing practice. J Issues Nurs 2011; 16: 8. 22088157

[7] Morley G Ives J Bradbury-Jones C , et al. What is ‘moral distress’? a narrative synthesis of the literature. Nurs Ethics 2019; 26: 646–662. 28990446 10.1177/0969733017724354 PMC6506903

[8] Epstein E Whitehead P Prompahakul C , et al. Enhancing understanding of moral distress: the measure of moral distress for health care professionals. AJOB Empir Bioeth 2019; 10: 113–124. 31002584 10.1080/23294515.2019.1586008

[9] Mehlis K Bierwirth E Laryionava K , et al. High prevalence of moral distress reported by oncologists and oncology nurses in end‐of‐life decision making. Psych‐Onc 2018; 27: 2733–2739. 10.1002/pon.4868 30156350

[10] Rodriguez-Ruiz E Campelo-Izquierdo M Veiras P , et al. Moral distress among healthcare professionals working in intensive care units in Spain. Med Intensiva 2022; 46: 383–391. 35753710 10.1016/j.medine.2021.06.005

[11] Prentice T Janvier A Gillam L , et al. Moral distress in neonatology. An Pediatr 2021; 148: Article e2020031864. 10.1542/peds.2020-031864 34285081

[12] Giannetta N Villa G Pennestrì F , et al. Instruments to assess moral distress among healthcare workers: a systematic review of measurement properties. Int J Nurs Stud 2020; 111: 103767. 32956930 10.1016/j.ijnurstu.2020.103767

[13] Corley M Elswick R Gorman M , et al. Development and evaluation of a moral distress scale. J Adv Nurs 2001; 33: 250–256. 11168709 10.1046/j.1365-2648.2001.01658.x

[14] Hamric A Blackhall L . Nurse-physician perspectives on the care of dying patients in intensive care units: collaboration, moral distress, and ethical climate. Crit Care Med 2007; 35: 422–429. 17205001 10.1097/01.CCM.0000254722.50608.2D

[15] Hamric A Borchers C Epstein E . Development and testing of an instrument to measure moral distress in healthcare professionals. AJOB Prim Res 2012; 3: 1–9.26137345

[16] Whitehead P Herbertson R Hamric A , et al. Moral distress among healthcare professionals: report of an institution‐wide survey. J Nurs Scholarsh 2015; 47: 117–125. 25440758 10.1111/jnu.12115

[17] Fujii T Katayama S Miyazaki K , et al. Translation and validation of the Japanese version of the measure of moral distress for healthcare professionals. Health Qual Life Outcome 2021; 19: 1–11. 10.1186/s12955-021-01765-1 PMC8045393 33849571

[18] Fischer-Grönlund C Brännström M . The Swedish translation and cultural adaptation of the measure of moral distress for healthcare professionals (MMD-HP). BMC Med Ethics 2021; 22: 1–7. 34772400 10.1186/s12910-021-00722-3 PMC8588668

[19] Wocial L Weaver M . Development and psychometric testing of a new tool for detecting moral distress: the moral distress thermometer. J Adv Nurs 2013; 69: 167–174. 22607094 10.1111/j.1365-2648.2012.06036.x

[20] Wolf A White K Epstein E , et al. Palliative care and moral distress: an institutional survey of critical care nurses. Crit Care Nurse 2019; 39: 38–49. 31575593 10.4037/ccn2019645

[21] Sonis J Pathman D Read S , et al. A national study of moral distress among US internal medicine physicians during the COVID-19 pandemic. PLoS One 2022; 17: Article e0268375. 35576206 10.1371/journal.pone.0268375 PMC9109912

[22] Powell S Engelke M Swanson M . Moral distress among school nurses. J Sch Nurs 2018; 34: 390–397. 28425313 10.1177/1059840517704965

[23] Schneider J Hiebel N Kriegsmann-Rabe M , et al. Moral distress in hospitals during the first wave of the COVID-19 pandemic: a web-based survey among 3,293 healthcare workers within the German network university medicine. Front Psychol 2021; 12: 775204. 34867685 10.3389/fpsyg.2021.775204 PMC8636670

[24] Fischer- Grönlund C Brännström M Isaksson U . Psychometric testing of the Swedish version of the measure of moral distress for healthcare professionals (MMD-HP). BMC Med Ethics 2023; 24: 35. 37254086 10.1186/s12910-023-00916-x PMC10228011

[25] Epstein EG Shah R Marshall M . Effect of a moral distress consultation service on moral distress, empowerment, and a healthy work environment. HEC Forum 2021; 35: 21–35. 33811568 10.1007/s10730-021-09449-5

[26] World Health Organization . Process of translation and adaptation of instruments. Geneva: World Health Organization, https://www.coursehero.com/file/30372721/WHO-Process-of-translation-and-adaptation-of-instrumentspdf/ (2020, accessed 05-27 2020).

[27] Willis G Artino A Jr . What do our respondents think we’re asking? using cognitive interviewing to improve medical education surveys. J Grad Med Educ 2013; 5: 353–356. 24404294 10.4300/JGME-D-13-00154.1 PMC3771159

[28] Epstein J Santo R Guillemin F . A review of guidelines for cross-cultural adaptation of questionnaires could not bring out a consensus. J Clin Epidemiol 2015; 68: 435–441. 25698408 10.1016/j.jclinepi.2014.11.021

[29] Lützen K Kvist B . Moral distress: a comparative analysis of theoretical understandings and inter-related concepts. HEC Forum 2012; 24: 13–25. 22454155 10.1007/s10730-012-9178-9

[30] Af Sandeberg M Wenemark M Bartholdson C , et al. To change or not to change-translating and culturally adapting the pediatric version of the moral distress scale-revised (MDS-R). BMC Med Ethics 2017; 18: 14. 28219363 10.1186/s12910-017-0176-y PMC5319143

[31] Wocial L Ackerman V Leland B , et al. Pediatric ethics and communication excellence (PEACE) rounds: decreasing moral distress and patient length of stay in the PICU. HEC Forum 2017; 29: 75–91. 27815753 10.1007/s10730-016-9313-0

[32] Rushton C Kaszniak A Halifax J . A framework for understanding moral distress among palliative care clinicians. J Palliat Med 2013; 16: 1074–1079. 23777328 10.1089/jpm.2012.0490

[33] Semler L . Moral distress to moral success: strategies to decrease moral distress. Nurs Ethics 2022; 30: 58–70. 36259494 10.1177/09697330221114328

[34] Guttmann K Flibotte J Seitz H , et al. Goals of care discussions and moral distress among neonatal intensive care unit staff. J Pain Symptom Manag 2021; 62: 529–536. 10.1016/j.jpainsymman.2021.01.124 33516926

[35] Wocial L Slaven J Montz K , et al. Factors associated with physician moral distress caring for hospitalized elderly patients needing a surrogate decision-maker: a prospective study. J Gen Intern Med 2020; 35: 1405–1412. 32096085 10.1007/s11606-020-05652-1 PMC7210358

[36] Tian X Jin Y Chen H , et al. Instruments for detecting moral distress in clinical nurses: a systematic review. Inquiry 2021; 58: 1–12. 10.1177/0046958021996499 PMC8743918 33771048

